# Influence of Vitamin D Supplementation on Mental Health in Diabetic Patients: A Systematic Review

**DOI:** 10.3390/nu13113678

**Published:** 2021-10-20

**Authors:** Dominika Guzek, Aleksandra Kołota, Katarzyna Lachowicz, Dominika Skolmowska, Małgorzata Stachoń, Dominika Głąbska

**Affiliations:** 1 Department of Food Market and Consumer Research, Institute of Human Nutrition Sciences, Warsaw University of Life Sciences (WULS-SGGW), 159C Nowoursynowska Street, 02-776 Warsaw, Poland; 2 Department of Dietetics, Institute of Human Nutrition Sciences, Warsaw University of Life Sciences (WULS-SGGW), 159C Nowoursynowska Street, 02-776 Warsaw, Poland; aleksandra_kolota@sggw.edu.pl (A.K.); katarzyna_lachowicz@sggw.edu.pl (K.L.); dominika_skolmowska@sggw.edu.pl (D.S.); malgorzata_stachon@sggw.edu.pl (M.S.); dominika_glabska@sggw.edu.pl (D.G.)

**Keywords:** diabetes, supplementation, vitamin D, cholecalciferol, mental health, health-related quality of life (HRQOL), depression, anxiety, stress

## Abstract

Diabetes is associated with a number of mental health consequences, including enhanced risk of depression and anxiety, as well as decreased quality of life, and vitamin D deficiency is considered to be one of the factors that influence these outcomes in diabetic patients. The aim of the present study was to conduct a systematic review of the literature presenting the data regarding the influence of vitamin D supplementation on mental health in diabetic adults. This study was conducted in accordance with the Preferred Reporting Items for Systematic Reviews and Meta-Analyses (PRISMA) guidelines and registered in the International Prospective Register of Systematic Reviews (PROSPERO) database (Registration number CRD42020155779). A systematic search of the PubMed and Web of Science databases was performed, and the intervention studies published until September 2021 were included in the review. The human studies were included if an adult sample of diabetic individuals received vitamin D supplementation during the intervention and its effect on any mental health aspect was assessed, but studies presenting the influence of combined supplementation of multiple nutrients were excluded. After removing duplicate records, a total of 8514 publications were screened and assessed independently by two researchers, based on their title, abstract, and full text. Finally, six studies were included in the current systematic review, and the risk of bias was evaluated using the Newcastle–Ottawa Scale (NOS). The included studies analyzed the influence of a specific dose of vitamin D, or different doses of vitamin D, or compared the results of supplementation with a specific dose of vitamin D against the placebo group. The supplementation was performed for at least 12 weeks. The mental health outcomes analyzed in these studies included health-related quality of life, depression, anxiety, stress, and general mental health status of adult diabetic patients. The results of the majority of the studies confirmed the positive influence of vitamin D supplementation on the mental health of diabetic individuals. Those studies that analyzed the influence of vitamin D supplementation on depression and anxiety established the beneficial effect of the vitamin. In some studies, the influence of vitamin D supplementation on the health-related quality of life was not considered unless combined with mindfulness training. However, it must be emphasized that different dosage regimens and intervention periods were followed in the reviewed studies, and only a small number of studies were randomized against placebo, which should be considered as a limitation of the present study. The findings of the conducted systematic review demonstrated the positive influence of vitamin D supplementation on the mental health of diabetic patients, which was proved for anxiety and depression, but in the case of health-related quality of life, the positive effect was observed only when the intervention included mindfulness training. These outcomes suggest that supplementation should be recommended to improve the vitamin D status and the mental health of patients in this group.

## 1. Introduction

Diabetes comprises a group of metabolic diseases that are characterized by chronic hyperglycemia, which is caused by defects in insulin secretion (type 1 diabetes) and/or insulin action (type 2 diabetes), and which may result in lifelong consequences associated with damage to various organs and systems, resulting in complications such as diabetic retinopathy, nephropathy, and neuropathy, and various cardiovascular disorders [1]. Currently, it is one of the major causes of mortality and reduced life expectancy worldwide, while the global trends show an increasing rate of incidence, prevalence, death, and disability-adjusted life-years associated with diabetes, particularly for type 2 diabetes [2]. The World Health Organization (WHO) emphasizes that there is an urgent need to reverse this trend, and within global noncommunicable disease targets to be obtained by 2025, the WHO has indicated that the number of premature deaths related to diabetes should be reduced, as diabetes is responsible for about 1.5 million deaths every year [3].

Diabetes is also associated with other complications, including mental health problems. A systematic review and meta-analysis by Chireh et al. [4] and a meta-analysis by Rotella and Mannucci [5], who analyzed depression, established that diabetes is an independent risk factor for the onset of depressive symptoms and depression and that over 9.5 million of global depression cases are potentially attributed to diabetes. A similar association was observed between diabetes and enhanced risk of anxiety, as the systematic reviews and meta-analyses by Smith et al. [6] and Amiri and Behnezhad [7] showed that diabetes is associated with an increased incidence of elevated anxiety symptoms and anxiety disorders. In addition, the quality of life of diabetic patients is found to be reduced [8], and it is indicated by the systematic review and meta-analysis by Jing et al. [9] that a decrease in the quality of life in this group of patients is associated not only with diabetes but also with its consequences, including depression.

The effective management of diabetes includes following a proper and planned diet schedule [10], which must address existing micronutrients deficiencies, maintaining appropriate body weight, controlling blood glycemic, blood pressure, and lipid levels, and delaying or preventing the onset of complications related to prolonged diabetes [11]. Among various potential nutritional deficiencies, vitamin D deficiency is considered to be an important issue in diabetic patients, as a number of large observational studies have suggested an association between this deficiency and the onset of diabetes [12]. In addition, vitamin D deficiency is associated not only with an increased risk of diabetic retinopathy [13], but also with lower satisfaction with treatment and lower quality of life [14].

Nowadays, the role of vitamin D supplementation in improving the mental health of patients is extensively discussed, as this nutrient was proven to exert a positive effect on various components of mental health in the population of healthy individuals. The meta-analyses by Vellekkatt and Menon [15], Shaffer et al. [16], and Spedding [17] suggested that supplementation of vitamin D may be beneficial in reducing the symptoms of depression. Moreover, the meta-analysis by Cheng et al. [18] supported the positive effect of vitamin D supplementation on the alleviation of negative emotions, while a systematic review by Hoffmann et al. [19] revealed its role in improving the quality of life of diabetic patients.

Taking into account the serious mental health problems generally observed in diabetic individuals, combined with vitamin D deficiency in this group, as well as the potential therapeutic value of vitamin D supplementation, which was proven for healthy individuals, there is an urgent need to determine whether vitamin D supplementation may reduce mental health problems in diabetic individuals. The aim of the present study was to conduct a systematic review of the literature presenting data regarding the influence of vitamin D supplementation on mental health status in diabetic adults.

## 2. Materials and Methods

### 2.1. The Registration and Design

The systematic review was conducted in accordance with the Preferred Reporting Items for Systematic Reviews and Meta-Analyses (PRISMA) guidelines and registered in the International Prospective Register of Systematic Reviews (PROSPERO) database (Registration number CRD42020155779). Studies that analyzed the association between vitamin D and mental health outcomes in diabetic patients were selected for the review [20]. The procedures adopted in this study, including a systematic literature search, screening of the literature, inclusion of the studies, and reporting of results, were similar to those applied in previous research works [21] and were in agreement with the PRISMA guidelines [22].

A literature search of the PubMed and Web of Science databases was performed, and intervention studies published until September 2021 were included. The search was conducted in two stages: in the first stage, studies published till October 2019 were searched, and in the second stage (supplementary stage), studies published from October 2019 to September 2021 were reviewed.

### 2.2. The Assessment of Eligibility and Inclusion

The studies that analyzed the influence of vitamin D supplementation on the mental health of diabetic patients were eligible to be included in the analysis.

The inclusion and exclusion criteria are presented in Table 1. No other inclusion or exclusion criteria pertaining to the type of diabetes, nature of the studied population, or country were taken into consideration in the present analysis.

### 2.3. The Systematic Review Procedure

The applied electronic search strategy for PubMed and Web of Science databases is presented in Supplementary Table S1.

**Figure 1:**
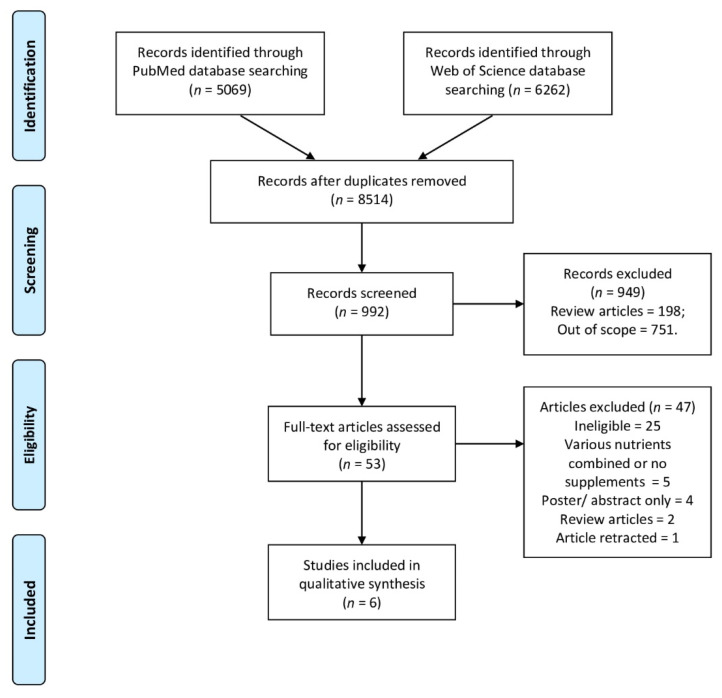
The identification, screening, eligibility, and inclusion within the systematic review.

The identification, screening, eligibility, and inclusion within the systematic review are presented in Figure 1. The identification of the studies and verification of the results were performed by two independent researchers simultaneously, and the search was performed in three stages based on the title, abstract, and full text. At each stage, in case of any disagreement between the examiners, the opinion of the third researcher was taken. If the full texts of eligible articles were not available, they were obtained from the corresponding author of the study.

### 2.4. The Procedure of Data Extraction

The data were extracted by two independent researchers simultaneously, and the results of the extracted data were further verified. Any disagreement between them was resolved by taking the suggestions of the third researcher. If any information was not available in the selected articles, it was obtained from the corresponding author of the study (such information is referred to within the study as data provided on request).

The authors extracted information about the characteristics of the study, which are as follows:

(1) Basic details of the studies included in the systematic review, which include the design of the study, country/location, nature of the study group, and time;
(2) Basic characteristics of the study participants, which include the number of participants and of female participants, age, and inclusion/exclusion criteria;
(3) Basic description regarding the exposure and outcomes assessed in the included studies, which include vitamin D assessment, vitamin D supplementation, mental health outcome, and psychological measures;
(4) Findings of the included studies, including observations and conclusions.

The risk of bias, namely the methodological quality of the studies [23] was assessed using Newcastle–Ottawa Scale (NOS) [24]. On the basis of the NOS criteria, the following parameters of the study were assessed: selection (scored 0–4), comparability (scored 0–2), and exposure/outcome (scored 0–3). Finally, based on the total score awarded, the studies were categorized as follows: very high risk of bias (total score: 0–3), high risk of bias (total score: 4–6), and low risk of bias (total score: 7–9) [25]. 

## 3. Results

The basic details of the studies included in the systematic review [26,27,28,29,30,31] are presented in Table 2. The included studies that evaluated the influence of vitamin D supplementation on the mental health of diabetic patients were conducted mainly in Iran [29,30,31], and also in the United States of America [28], Canada [27], and the Netherlands [26]. The study sample comprised patients recruited from the specific clinics of the hospitals [27,29,31] or from general practitioners [26]. Some groups were recruited to be characterized by specific diabetes complications, such as chronic kidney disease [27] or painful diabetic neuropathy [31]. Some studies included patients suffering from mental health problems, such as anxiety [29] or depressive symptomatology [28,30], while some included patients were diagnosed with vitamin D deficiency only [29,31].

The basic characteristics of the participants of the studies included in the systematic review are presented in Table 3. The studies included in this systematic review were conducted mainly in small samples of participants (less than 100 individuals) [28,29,30] or larger samples comprising more than 100 [26,27] or 200 individuals [31], while some studies were performed in smaller but homogenous samples comprising exclusively female individuals [28,29]. The inclusion criteria were based mainly on the diagnosis of type 2 diabetes mellitus [26,28,29,30,31], type 1 or type 2 diabetes mellitus [27], a specified clinical condition, and applied treatment, as well as the presence of specific diabetes complications, such as chronic kidney disease [27] or diabetic neuropathy [31].

The basic description of the exposure and outcomes within the studies included to the systematic review is presented in Table 4. The included studies analyzed the influence of a specific dose of vitamin D [28] or different doses of vitamin D [27], or compared the influence of a specific dose of vitamin D with the placebo group [26,29,30,31]. The supplementation of vitamin D was performed for a minimum of 12 weeks [31], while in the majority of the studies, the supplementation period was 6 months [26,27,28]. The mental health outcomes evaluated in these works were mainly health-related quality of life [26,27,31], depression [28,29,30], anxiety [28,29], stress [29], or general mental health status [28].

The findings presented within the studies included in the systematic review are presented in Table 5. Most of the studies demonstrated the beneficial influence of the applied vitamin D supplementation on mental health [28,29,30,31] and concluded that vitamin D shows therapeutic [28,29,31] or preventive effect [30]. Only two studies did not confirm the positive impact of vitamin D supplementation [26,27].

The summary of findings presenting association between vitamin D supplementation and mental health in diabetic adults, accompanied by the Newcastle–Ottawa Scale (NOS) score for the studies included to the systematic review are presented in Table 6. The vast majority of the included studies (five studies among the six studies) were identified as high-quality studies with a low risk of bias [26,27,28,29,31], but only four were randomized against placebo [26,29,30,31], and three of them were randomized against placebo and possessed low risk of bias [26,29,31]. Only two studies did not provide conclusion for the influence of vitamin D supplementation on the assessed mental health outcomes, namely health-related quality of life [26,27]. However, a positive influence was observed in one study that analyzed the effect of supplementation combined with mindfulness training on the health-related quality of life [31]. All the studies that analyzed the influence of vitamin D supplementation on depression [28,29,30] and anxiety [28,29] confirmed the beneficial effect of vitamin D on improving mental health.

## 4. Discussion

The results of the studies included in the systematic review demonstrate that vitamin D supplementation may be beneficial in diabetic patients for the studied components of mental health. As indicated previously, diabetes may be associated with deteriorating mental health, which was proven for depression [4,5], anxiety [6,7], and quality of life [8,9]. Hence, vitamin D supplementation is an efficient approach to treat these psychological disorders in particular and may be considered as a promising therapeutic strategy for the prevention and treatment of mental health problems in general practice.

Although the number of studies that analyzed the influence of vitamin D supplementation on the mental health of diabetic patients is small, the results are consistent, and only two studies did not confirm the positive effect of vitamin D on the health-related quality of life [26,27]. However, it should be emphasized that a study by Westra et al. [26], assessed the influence of a supplemented dose applied every month (participants received 1250 µg of vitamin D or placebo once a month), and a study by Mager et al. [27], compared the effect of the supplemented dose administered daily and monthly (50 µg/day and 1000 µg/month), which may have influenced the observed results of the studies. The results of the studies comparing the effect of vitamin D supplementation depending on the time intervals and dosage regimens, and hence comparing the effect of daily and monthly doses, provide contradictory results. The studies that assessed equivalent cumulative doses revealed that daily supplementation of vitamin D is more effective than weekly, and monthly administration is the least effective approach [32], or that monthly administration is more effective than daily [33]. Despite the fact that monthly administration can improve adherence in patients, while being quite safe and not causing side effects or toxicity [34], it is still not evident how such a regimen may influence mental health. The single study that verified the influence of daily and monthly supplementation of vitamin D on the mental health of diabetic patients revealed that both the regimens presented comparable results for the quality of life, but only modest improvement was observed [27].

The other potential reason for no significant impact of vitamin D supplementation on the mental health of diabetic patients when the health-related quality of life was assessed [26,27] may be associated with the assessed mental health outcome, as for depression [28,29,30] and anxiety [28,29] all the studies confirmed the beneficial effect of vitamin D. The health-related quality of life is associated not only with the mental component but is also related to the symptoms of the disease or health condition of the patient, treatment side effects, and functional status across physical, social, and mental health domains [35]. Therefore, it is indicated that for diabetic patients, common complications that influence the health-related quality of life are coronary arterial disease followed by renal failure, blindness, and a combination of micro- and macrovascular complications, and also, according to some studies, sexual dysfunction [36]. It indicates that for diabetic patients, physical well-being may be more important than mental well-being, as the health-related quality of life may be affected by various complications of the disease which directly may reduce the quality of life. However, psychiatric disorders, especially depression, are still predominant factors that negatively affect the health-related quality of life in diabetic patients, but globally they may not be the most crucial influencing factor in this group of patients [37].

The suggested complicated effect of vitamin D supplementation on the health-related quality of life in diabetic patients and no proven improvement of all mental health conditions after vitamin D supplementation may be confirmed by the observation that supplementation provided beneficial effects when it was combined with mindfulness training [31]. Mindfulness, defined as awareness and nonjudgmental acceptance of a patient’s moment-to-moment experience, is indicated to provide relief from psychological distress [38]. In addition, especially for diabetic patients, it may modify the influence of physical determinants on health-related quality of life by accepting and learning to live with numerous complications of diabetes. Moreover, some studies report that the mindfulness approach may contribute to better glucose regulation, since patients are less likely to develop obesity and show a greater sense of control [39], which may further enhance their health-related quality of life.

While for the health-related quality of life, the results were ambiguous, for depression [28,29,30] and anxiety [28,29], vitamin D supplementation was stated to improve the mental health outcomes in diabetic patients in all the studies. Such observations are promising, as it is proven that the prior presence of depression [40] or anxiety is associated with worse outcomes of therapy of diabetes [41]. Thus, effective reduction of the symptoms of depression and anxiety may also improve the effectiveness of antidiabetic treatment. 

Furthermore, vitamin D supplementation should not be the only approach to improve the mental health of diabetic patients and should be accompanied by cognitive therapy for effective results, especially a mindfulness-based one, which is expected to reduce the depression symptoms [42] and also improve the health-related quality of life, as indicated above. 

While the current recommendations for prevention and treatment of depression stress the need to identify high-risk groups, diabetic patients should constitute one of such groups [43], so all possible actions to improve the mental well-being of those patients should be implemented. Although the exact mechanism of action of vitamin D is still unknown and numerous important questions pertaining to its influence on mental health outcomes remain unanswered [44], diabetic patients may still benefit from its positive influence and should receive adequate supplementation to treat vitamin D deficiency and to improve their mental health.

Though the current review provides novel and interesting observations, it has certain limitations. It must be emphasized that different dosage regimens and intervention periods were adopted for supplementation, and hence the results may be incomparable. In spite of the fact that during the literature search, broad criteria were applied to select the studies (to not miss any potentially eligible study), the topic of the influence of vitamin D supplementation on the mental health of diabetic patients is still novel and has not been studied by many research teams until now. Moreover, only four studies were randomized against placebo [26,29,30,31], so it must be indicated that the results of the other studies were not so strong in spite of being assessed using NOS, as characterized by the low risk of bias. The most important contributions to this field were made by three studies randomized against placebo, and associated with a low risk of bias [26,29,31]. However, their results may be perceived as contradictory, as one of them did not support the positive influence of vitamin D on the health-related quality of life of diabetic patients [26]. Based on the previous observations that the health-related quality of life is a complex parameter and that it is associated not only with mental components but also with other symptoms of a disease or health condition [35], it may still influence the final conclusions of the analysis.

## 5. Conclusions

The conducted systematic review confirmed the positive influence of vitamin D supplementation on the mental health of diabetic patients, which was evident for anxiety and depression, but for health-related quality of life, supplementation should be combined with mindfulness training to obtain beneficial results. All the above-mentioned observations support the notion that vitamin D supplementation should be recommended to improve the vitamin D status and mental health outcomes in this group of patients.

## Figures and Tables

**Table 1:** The inclusion and exclusion criteria for the systematic review.

Criteria	
Inclusion	(1) studies published in peer-reviewed journals; (2) full text articles published in English; (3) studied group of adults; (4) diabetes mellitus diagnosed in the studied population; (5) supplementation of vitamin D applied; (6) assessed outcome including any component of mental health assessed while using any method (either subjective or objective)
Exclusion	(1) animal model studies; (2) studies conducted in participants with any intellectual disabilities; (3) studies conducted in participants with any eating disorders; (4) studies conducted in participants with any neurological disorders (Alzheimer’s disease, epilepsy, etc.); (5) studies including assessment of combined supplementation of multiple nutrients.

**Table 2:** The basic details of the studies included to the systematic review.

Ref.	Authors, Year	Design of the Study	Country/Location	Studied Group	Time
[26]	Westra et al. 2016	Randomized, double-blind, placebo-controlled trial—SUNNY trial (StUdy the effect of vitamiN D supplemeNtation on glYcaemic control in type 2 DM)	Netherlands/in and around the city of Alkmaar	Adults with type 2 diabetes mellitus derived from general practitioners	July 2012 to April 2013
[27]	Mager et al., 2017	Open-label randomized clinical trial	Canada/Northern Alberta	Adults with diabetes mellitus and chronic kidney disease from two clinics (Diabetes Nephropathy Prevention Clinic and Renal Insufficiency Clinic in the Northern Alberta)	2011 to 2014
[28]	Penckofer et al., 2017	Open-label, proof-of-concept study—Sunshine Study	United States of America/Chicago *	Women with type 2 diabetes mellitus and significant depressive symptomology	From October 2009 to May 2012 *
[29]	Fazelian et al., 2019	Randomized double-blind placebo-controlled clinical trial	Iran/Shahr-e-Kord	Women with type 2 diabetes mellitus, vitamin D deficiency, and anxiety recruited from Shahr-e-Kord Diabetes Clinic	Not specified
[30]	Omidian et al., 2019	Randomized placebo-controlled double-blind clinical trial	Iran	Patients with type 2 diabetes mellitus and mild to moderate depressive symptoms	Inclusion from October 2017 to May 2018 and follow up for 12 weeks
[31]	Davoudi et al., 2021	Randomized placebo-controlled clinical trial	Iran/Kermanshah	Patients with painful diabetic neuropathy and vitamin D deficiency referred to Beheshti hospital, Kermanshah, Iran	September 2019 to January 2020

* data provided on request; DM—diabetes mellitus.

**Table 3:** The basic characteristics of the participants of the studies included in the systematic review.

Ref.	Number of Participants (Female Participants) Age (Mean/Median; SD/Range)	Inclusion Criteria	Exclusion Criteria
[26]	187 (62) According to group: 67.0 ± 8.0 years for vitamin D supplementation group 68.0 ± 9.0 years for placebo group	Inclusion: aged ≥18 years; type 2 diabetes mellitus; treated with lifestyle advice, metformin, and/or sulfonylurea derivatives; serum HbA1c stable and ≤8.0% for the last three months without recent changes in hypoglycemic agents	Exclusion: insulin therapy; impaired renal function (GFR < 30 mL/min/1.73 m^2^); any granuloma forming disorder; hypercalcemia (serum calcium > 2.65 nmol/L); serum 25-hydroxyvitamin D (25(OH) D) <15 nmol/L or >150 nmol/L; urolithiasis; psychiatric treatment for schizophrenia; organic mental disorder or bipolar disorder currently or in the past; insufficient knowledge of the Dutch language; substance abuse (other than nicotine); increase in HbA1c to >8.5% within the study; hypersensitivity to cholecalciferol or placebo within the study; onset of urolithiasis within the study; any change in antidiabetic medication within the study; serum 25(OH)D <15 or >250 nmol/L within the study; taking vitamin D supplements other than planned within the study
[27]	120 (44) 65.32 (58.8–68.8) *	Inclusion: aged 18–80 years; type 1 or type 2 diabetes mellitus; stages 1–4 of chronic kidney disease (GFR 15–89 mL/min/1.73 m^2^)	Exclusion: co-morbid conditions known to affect vitamin D metabolism including gastrointestinal, liver, rheumatoid, or bone disorders (e.g., hyperthyroidism, untreated celiac disease, cancer, Paget’s disease, sarcoidosis, malabsorption); severe, permanent vision impairment; pregnancy; body mass of >136 kg; drug therapy known to interfere with vitamin D (e.g., oral glucocorticoids, cholestyramine, colestipol, mineral oil, Orlistat, digoxin); applying other forms of active vitamin D metabolites (e.g., calcitriol, vitamin D2); dialysis therapy or being on a kidney transplant list; pre-existing hypercalcemia (>2.75 mmol/L), hyperphosphatemia (>2.0 mmol/L), severe secondary hyperparathyroidism (PTH > 66 pmol/L), and serum 25(OH)D > 200 nmol/L; serum 25(OH)D < 37.5 nmol/L at time of screening; undergoing strict heavy exercise for weight control; using sunscreen lotion on a daily basis
[28]	50 (50) 54.3 ± 10.6	Inclusion: women; aged ≥18; medically stable type 2 diabetes mellitus with HbA1c ≤9%; elevated depressive symptoms measured using the CES-D Scale; average score of ≥16 on the CES-D Scale from two screenings (phone and baseline) within 4 weeks of each screening	Exclusion: taking vitamin D supplementation during 2 months prior to enrolment; vitamin D levels ≥80 nmol/L; malabsorption problems (e.g., Crohn’s disease, celiac sprue) and/or bariatric surgery; hypercalcemia; severe complications of diabetes (e.g., amputation, blindness); low thyroid function; pregnancy; active suicidal ideation; history of bipolar depression; psychotic disorders; current alcohol or substance disorders
[29]	51 (51) According to group: 48.5 ± 7.6 years for vitamin D supplementation group 46.3 ± 11.2 years for placebo group	Inclusion: women; aged 20–60 years; diagnosis of type 2 diabetes mellitus based on the World Health Organization guidelines; mild, moderate, or severe anxiety; vitamin D deficiency or insufficiency (serum 25(OH)D of 25–75 nmol/L)	Exclusion: neurological or psychiatric disorders; taking any medications for depression or vitamin D/multivitamin supplements during the last 4 months; alcohol consumption; pregnancy; lactation
[30]	66 (27) According to group: 49.7 ± 6.5 years for vitamin D supplementation group 51.3 ± 5.9 years for placebo group	Inclusion: aged 30–60 years; type 2 diabetes mellitus; BMI 20–30 kg/m^2^; willingness to maintain the current diet, physical activity, and lifestyle for 3 months	Exclusion: receiving herbal products or dietary supplements for 3 months before and throughout the intervention; major depressive disorder and taking antidepressants; chronic kidney diseases; hepatobiliary diseases; gastrointestinal diseases; taking drugs that interact with vitamin D such as anticonvulsant drugs; using insulin or thiazolidinediones or anti-obesity drugs; pregnancy; lactation; any changes in the type or dosage of medications during the study; lack of adherence to the trial based on refusing to consume at least 90% of recommended treatments
[31]	204 (92) According to group: 56.3 ± 9.9 years for placebo group 53.3 ± 8.9 years for placebo + mindfulness group 54.8 ± 9.4 years for mindfulness group 54.5 ± 9.0 years for vitamin D supplementation group 56.6 ± 9.8 years for vitamin D supplementation + mindfulness group	Inclusion: age 20–70 years; type 2 diabetes mellitus; neuropathy; vitamin D insufficiency or deficiency (serum 25(OH)D of 25–75 nmol/L)	Exclusion: major co-morbid disease (e.g., coronary heart disease, psychiatric or neurological diseases); taking vitamin D or any multivitamins during the last three months; using any substance and drinking alcohol; pregnancy; more than one absence in mindfulness sessions

* data provided on request; BMI—body mass index; CES-D—Center for Epidemiologic Studies Depression Scale; GFR—glomerular filtration rate; HbA1c—hemoglobin A1c (glycated hemoglobin); PTH—parathyroid hormone (parathormone).

**Table 4:** The basic description of the exposure and outcomes within the studies included to the systematic review.

Ref.	Vitamin D Assessment	Vitamin D Supplementation	Mental Health Component	Psychological Measure
[26]	25(OH) vitamin D level in blood Sun exposure	1250 µg/month vs. placebo for 6 months	Health-related quality of life (HRQOL)	The Dutch version of the Short Form 36 (SF-36)
[27]	25(OH) and 1,25(OH)2 vitamin D level in blood, 3-day food record	50 µg/day vs. 1000 µg/month for 6 months	Health-related quality of life (HRQOL)	Short Form 36 (SF-36)
[28]	25(OH) vitamin D level in blood	1250 µg/week for 6 months	(1) depression (2) anxiety (3) mental health status	(1) Center for Epidemiologic Studies Depression (CES-D) Scale; Patient Health Questionnaire-9 (PHQ-9) (2) State–Trait Anxiety Inventory (STAI) (3) Short Form 12 (SF-12)
[29]	25(OH) vitamin D level in blood, 3-day food record, Sun exposure	1250 µg/2 weeks vs. placebo for 16 weeks	Depression, anxiety, stress	Depression, Anxiety, and Stress Scales (DASS-21) questionnaire
[30]	25(OH) vitamin D level in blood	100 µg/day vs. placebo for 3 months	Depression	Persian version of Beck Depression Inventory-II (BDI-II-PERSIAN)
[31]	25(OH) vitamin D level in blood, 3-day food record, Sun exposure	100 µg/day vs. placebo for 12 weeks	Health-related quality of life (HRQOL)	The Neuropathy Specific Quality of Life questionnaire (NeuroQol)

**Table 5:** The findings presented within the studies included to the systematic review.

Ref.	Observations	Conclusions
[26]	A small significant difference (adjusted β: −8.90; 95% CI: −17.16 to −0.65) between both groups was seen concerning the SF-36 domain role limitations due to physical problems in disadvantage of the vitamin D group.	Six months of vitamin D supplementation did not improve HRQOL in non-vitamin D-deficient people with type 2 diabetes mellitus managed on oral antidiabetic therapy.
[27]	No significant differences over six months between groups were observed in quality of life measures (*p* > 0.05).	Daily (50 µg/day) and monthly (1000 µg/month) vitamin D3 supplementation for six months in adults with diabetes mellitus and CKD was safe, and resulted in equivalent adherence and improvements in overall vitamin D status, but only modest changes in quality of life (no significant changes).
[28]	There was a significant decrease in depression (CES-D and PHQ-9, *p* < 0.001) and anxiety (state and trait, *p* < 0.001). An improvement in mental health status (SF-12, *p* < 0.001) was also found. After controlling for covariates (race, season of enrollment, baseline vitamin D, baseline depression (PHQ-9), and body mass index), the decline in depression remained significant (CES-D, *p* < 0.001).	This proof-of-concept study found that weekly administration of 1250 µg of vitamin D in women with type 2 diabetes mellitus who had significant depressive symptoms and low 25(OH)D levels had an improvement in depression, anxiety, and mental health outcomes.
[29]	Anxiety score changes were significantly lower in vitamin D group than the controls (*p* = 0.001). Within-group comparison indicated that depression in supplement group with lower vitamin D levels was significantly reduced.	Vitamin D supplementation can improve mood status in female diabetics with anxiety and vitamin D deficiency.
[30]	BDI-II scores decreased from 15.2 ± 9.6 to 9.8 ± 7.2 (*p* < 0.001) in the vitamin D group and 15.5 ± 11.2 to 13.7 ± 11.5 (*p* = 0.03) in placebo group. This decrease in BDI-II scores were significant (27.6 vs. 10.8%) compared with placebo (*p* = 0.02).	Supplementation of vitamin D in type 2 diabetes mellitus patients may protect patients against the onset of major depressive disorder.
[31]	At the end-of-treatment, results showed improvement in all groups except the “placebo only” group for outcome variables. There was no difference between vitamin D and mindfulness groups (within and not combined with placebo) in posttest. However, “vitamin D + mindfulness” has a greater improvement rather than vitamin D and mindfulness groups (*p* < 0.05).	Combining vitamin D and mindfulness training can reduce pain severity and pain-related disability, so with these changes, patients experience improvement in their quality of life.

BDI-II—Beck Depression Inventory-II; CES-D—Center for Epidemiologic Studies Depression; CI—confidence interval; CKD—chronic kidney disease; HRQOL—Health-related quality of life; PHQ-9—Patient Health Questionnaire-9; SF-12—Short Form 12; SF-36—Short Form 36.

**Table 6:** The summary of findings presenting association between vitamin D supplementation and mental health in diabetic adults, accompanied by the Newcastle–Ottawa Scale (NOS) score for the studies included in the systematic review.

Ref.	Association between Vitamin D Supplementation and Mental Health in Diabetic Adults		Quality	
	Studied Outcome	Supporting/Not Supporting ^a^	Randomization against Placebo	Newcastle–Ottawa Scale (NOS) Score ^b^
[26]	Health-related quality of life (HRQOL)	Not supporting	+	9
[27]	Health-related quality of life (HRQOL)	Not supporting	-	9
[28]	Depression, anxiety, mental health status	Supporting	-	7
[29]	Depression, anxiety, stress	Supporting	+	8
[30]	Depression	Supporting	+	6
[31]	Health-related quality of life (HRQOL)	Supporting (for vitamin D combined with mindfulness training)	+	7

^a^ Supporting—positive influence of vitamin D supplementation on mental health; not supporting—no positive influence of applied vitamin D supplementation on mental health; ^b^ the Newcastle–Ottawa Scale (NOS) score attributed to following categories: very high risk of bias (0–3 NOS points), high risk of bias (4–6 NOS points), low risk of bias (7–9 NOS points) [24].

## Supplementary Materials

The following are available online at www.mdpi.com/article/10.3390/nu13113678/s1, Table S1: Supplementary Table S1. The applied electronic search strategy for PubMed and Web of Science databases.
